# Masitinib treatment in patients with progressive multiple sclerosis: a randomized pilot study

**DOI:** 10.1186/1471-2377-12-36

**Published:** 2012-06-12

**Authors:** Patrick Vermersch, Rabah Benrabah, Nicolas Schmidt, Hélène Zéphir, Pierre Clavelou, Cyrille Vongsouthi, Patrice Dubreuil, Alain Moussy, Olivier Hermine

**Affiliations:** 1 Department of Neurology, University of Lille Nord de France (EA2686), Hôpital Roger Salengro, CHU de Lille, Lille cedex, 59037, France; 2 Private Practice, Paris, France; 3 Private Practice, Rueil-Malmaison, France; 4 Hôpital Gabriel Montpied, Clermont-Ferrand, France; 5 Private Practice, Montpellier, France; 6 AB Science, SA, Paris, France; 7 Inserm U891, Centre de Recherche en Cancérologie de Marseille, Signalisation, Hematopoiesis and Mechanisms of Oncogenesis, Centre de référence des mastocytoses, Marseille, France; Institut PaoliCalmettes, Marseille, France, Université Méditerranée, Marseille, France; 8 Service d’hématologie adulte, Centre de référence des mastocytoses, CNRS UMR 8147, Hôpital Necker, Université Paris Descartes, 149 - 161 rue de Sèvres, Paris, 75743, France

**Keywords:** Multiple sclerosis, Primary progressive multiple sclerosis, Secondary progressive multiple sclerosis, Tyrosine kinase inhibitor, Masitinib, Mast cells

## Abstract

**Background:**

Treatment options for patients suffering from progressive forms of multiple sclerosis (MS) remain inadequate. Mast cells actively participate in the pathogenesis of MS, in part because they release large amounts of various mediators that sustain the inflammatory network. Masitinib, a selective oral tyrosine kinase inhibitor, effectively inhibits the survival, migration and activity of mast cells. This exploratory study assessed the safety and clinical benefit of masitinib in the treatment of primary progressive MS (PPMS) or relapse-free secondary progressive MS (rfSPMS).

**Methods:**

Multicenter, randomized, placebo-controlled, proof-of-concept trial. Masitinib was administered orally at 3 to 6 mg/kg/day for at least 12 months, with dose adjustment permitted in event of insufficient response with no toxicity. The primary response endpoint was the change relative to baseline in the multiple sclerosis functional composite score (MSFC). Clinical response was defined as an increase in MSFC score relative to baseline of > 100%.

**Results:**

Thirty-five patients were randomized to receive masitinib (N = 27) or placebo (N = 8). Masitinib was relatively well tolerated with the most common adverse events being asthenia, rash, nausea, edema, and diarrhea. The overall frequency of adverse events was similar to the placebo group, however, a higher incidence of severe and serious events was associated with masitinib treatment. Masitinib appeared to have a positive effect on MS-related impairment for PPMS and rfSPMS patients, as evidenced by an improvement in MSFC scores relative to baseline, compared with a worsening MSFC score in patients receiving placebo; +103% ± 189 versus -60% ± 190 at month-12, respectively. This positive, albeit non-statistically significant response was observed as early as month-3 and sustained through to month-18, with similar trends seen in the PPMS and rfSPMS subpopulations. A total of 7/22 (32%) assessable masitinib patients reported clinical response following 12 months of treatment (according to the modified intent-to-treat population, observed cases) compared with none in the placebo group. The Expanded Disability Status Scale remained stable for both treatment groups.

**Conclusion:**

These data suggest that masitinib is of therapeutic benefit to PPMS and rfSPMS patients and could therefore represent an innovative avenue of treatment for this disease. This exploratory trial provides evidence that may support a larger placebo-controlled investigation.

## Background

Multiple sclerosis (MS) is an inflammatory condition that damages the myelin of the central nervous system, leading to neurologic impairment and possibly severe disability. MS is characterized by chronic patchy inflammation of the central nervous system with demyelination and gliosis (scarring). It is thought that progression of lesions in MS might have two components: an active immunological aspect and a degenerative aspect, although it is unknown to what extent these are causally interrelated. Four principal courses of MS are currently defined according to clinical characteristics; namely: Relapsing Remitting MS (RRMS), Secondary Progressive MS (SPMS), Primary Progressive MS (PPMS), and Progressive Relapsing MS (PRMS). The disease typically presents as RRMS, with more than 50% of RRMS patients entering a progressive phase (SPMS) following a highly variable delay [1]. Approximately 10 to 15% of patients present with PPMS, which is characterized by continuous disease progression from the onset of disease, i.e. without relapses and remissions, for which prognosis is considered as poor due to the relatively rapid development of advanced disability as compared with RRMS [2,3]. In general, drugs used in the treatment of MS are considered to act as immunomodulators, with the aim to decrease relapse rate, modify relapses, and diminish the accumulation of disability over time. Despite these approved therapies, many of which require parenteral administration, the unmet medical need in MS treatment remains substantial, especially for the subpopulations of PPMS and relapse-free SPMS (rfSPMS) for which there are currently no treatments proven to slow disease progression.

Masitinib mesilate, the investigatory drug of the present study, is a selective tyrosine kinase inhibitor that is particularly efficient in controlling the survival, migration and degranulation of mast cells (and thus indirectly controlling the array of proinflammatory and vasoactive mediators these cells can release), through inhibition of essential growth and activation signaling pathways [4]. Indeed, promising results have been reported from human clinical trials of masitinib in neurological and inflammatory disorders such as Alzheimer’s disease, rheumatoid arthritis, asthma and mastocytosis [5-8]. Several findings support the hypothesis that mast cells, which are found on both sides of the blood–brain barrier (BBB) [9-11], actively participate in the pathogenesis of MS and also experimental allergic encephalomyelitis (EAE), an animal model of human demyelinating diseases [11-14]. To this end, the ability and effect of masitinib in the inhibition of mast cell function in MS was explored using an EAE murine model considered to be a model for all progressive forms of MS (see Additional file 1: Preclinical data of masitinib in EAE). In summary, treatment of mice with masitinib led to a significant reduction in disease relative to control mice. A masitinib dose-dependent effect was also evident. Thus, molecules able to inhibit the survival and/or activation of mast cells may be able to control the symptoms and progression of MS or any related disease.

An exploratory study to assess the safety and clinical benefit of masitinib in the treatment of PPMS and rfSPMS patients was performed to investigate the hypothesis that masitinib’s targeted inhibitory action on mast cells may reduce the symptoms and progression of MS as compared with a placebo.

## Methods

### Study design and eligibility criteria

This was a multicenter, double-blind, randomized placebo-controlled, exploratory phase 2a study of masitinib in patients with PPMS or rfSPMS, treated over 12 months, with an extension phase possible. Patients were randomized to receive placebo or masitinib at an initial dose of 3 or 6 mg/kg/day, administered orally in two daily intakes. A centralized randomization schedule for packaging and labeling was generated and held by a third-party service (Cardinal Systems, Paris, France). All participants and study personnel were blinded to treatment allocated over the study’s duration. For each patient, all efficacy and safety parameters were recorded on the first day of treatment (baseline), with monthly patient visits scheduled for the first 3 months followed by visits once every 3 months thereafter for the duration of treatment. This study was approved by the local central ethics committee (Comité de Protection des Personnes Ile-de-France II) and complied with the Declaration of Helsinki. Written informed consent was obtained from all patients.

Patients aged 18 to 60, suffering from PPMS or rfSPMS as diagnosed by the ‘McDonald criteria’ [15,16] and having an Expanded Disability Status Scale (EDSS) score [17] between 2 to 6.5 with a progression > 1 within 2 years prior to inclusion, were eligible for this study. The following conditions were exclusion criteria: patients having SPMS with relapse in the 2 years before inclusion; treatment with interferon, glatiramer, oral or systemic corticosteroids, adrenocorticotropic hormone, or an investigational agent within 4 weeks of inclusion; and inadequate organ function defined via blood test levels.

### Study drug

Masitinib and placebo were supplied as 100 or 200 mg non divisible coated tablets (AB Science, France). Composition and dispensing of the masitinib and placebo treatments were identical except for the amount of active ingredient contained. Blinded dose adjustments of 1.5 mg/kg/day were permitted in the event of lack of response and manageable toxicity. Following predetermined criteria, treatment could be temporarily interrupted and/or the dosage decreased by 1.5 mg/kg/day in the event of toxicity. To manage possible cutaneous rash a mandatory concomitant treatment of cetirizine at 10 mg/day was administered for the first 30 days of treatment. Other permitted concomitant medications included analgesic without anti-inflammatory action and oral narcotic analgesic, although these were not to be taken on the day of a study visit until all efficacy evaluations were completed. Physical therapy, if performed at the time of study entry, was provided under a stable and consistent regimen. The following treatments were prohibited for the duration of the study: administration of immunomodulating; immunosuppressing; chemotherapy; paracetamol; and oral or parenteral concomitant corticosteroids, except in the event of protocol-defined demyelinating event for which methylprednisolone at 1 g/day for 3 days was permitted.

### Efficacy and safety assessment

Evaluation of treatment effect was based upon change in clinical neurological functions. The primary endpoint was the average change in multiple sclerosis functional composite (MSFC) score [18] relative to baseline, with clinical response defined as a >100% improvement (increase) from baseline.

The MSFC score is a multidimensional, MS-specific outcome measure, comprising of a timed 25-foot walk (T25FW) test measuring leg function and ambulation, a nine ­hole peg test (9-HPT) measuring arm and hand function and a Paced Auditory Serial Addition Test 3 seconds (PASAT-3”) measuring cognitive function. The MSFC was calculated as described in the National Multiple Sclerosis Society MSFC administration and scoring manual [19]. Secondary endpoints included analysis of the MSFC subcategories (namely, T25FW, 9-HPT and PASAT-3”), and the expanded disability status scale (EDSS) [17]. Safety was assessed throughout the study via physical examinations, vital signs, clinical laboratory evaluations and monitoring of adverse events (AEs), with all AEs recorded regardless of causality.

The study design originally allowed for analysis according to initial dose regimens, however a study amendment closed the 3.0 mg/kg/day treatment arm when it became apparent that insufficient response was observed at this dose (regardless of treatment being received), and that dose increases had been necessary for the vast majority of patients entering the 3.0 mg/kg/day initial dose group. Patients were therefore effectively pooled into a single population receiving masitinib at a dose of 6.0 mg/kg/day.

### Statistical analysis

Response analyses were performed on a modified intent-to-treat (mITT) population, defined as all randomized patients who received at least one dose of masitinib (i.e. the intent-to-treat population) and who had undergone baseline assessment and at least one post-baseline assessment of efficacy. Analysis was conducted according to two possible datasets: the last observation carried forward (LOCF) methodology (i.e. imputation of missing values) was performed for all efficacy endpoints other than MSFC response rates, which was based on those patients having relevant data at the given time point (i.e. observed cases). Descriptive statistics were used to analyze the safety profile of the study population at the time of study unblinding, after which date all placebo treated patients were withdrawn and only masitinib patients were able to continue treatment. Quantitative variables were compared using a nonparametric Wilcoxon rank sum test, and the Fisher’s exact test was used for comparing categorical variables.

## Results

### Baseline characteristics and patient disposition

**Figure 1:**
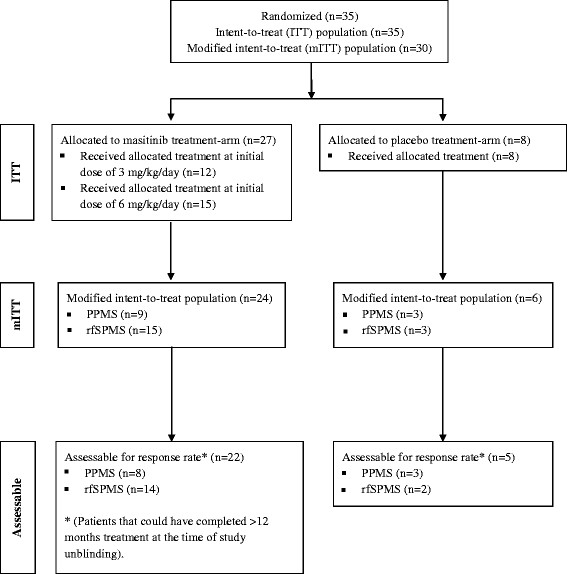
Trial profile.

Figure 1 shows the trial profile. Thirty-five patients were recruited and randomized from six centers in France, corresponding to an intent-to-treat (ITT) population of 27 patients in the masitinib group and 8 patients in the placebo group. Of these, 12 patients in the masitinib group started treatment on 3 mg/kg/day before being switched to 6 mg/kg/day (median time to switch was 2 months). Overall, patient baseline characteristics were well balanced between the masitinib and placebo groups (Table 1), as well as between the PPMS and rfSPMS subpopulations (data not shown). The mean MSCF score at baseline was slightly higher in the placebo group indicating better patient function as compared with the masitinib group. As expected, the duration of disease was longer in the rfSPMS population (median of 12.3 years) as compared with the PPMS population (median of 2.3 years). A total of 30 patients were eligible for the modified intent-to-treat (mITT) population consisting of 24 patients in the masitinib group (9 patients with PPMS and 15 patients with rfSPMS) and 6 patients in the placebo group (3 patients each with PPMS or rfSPMS). Of the five patients ineligible for the mITT population, two had not undergone any post-baseline efficacy assessment and three did not have a baseline PASAT-3” assessment performed (see Figure 1). A total of 27/30 patients (90%) from the mITT population, of which 22 patients received masitinib treatment and 5 patients received placebo, could have completed at least 12-months of treatment at the time of study unblinding, giving an assessable population for response rate at month-12 of 27 patients. Three patients were excluded from the response rate assessable population because they were still on-going at the time of unblinding but had received less than 12 months treatment.

**Table 1:** Baseline characteristics (ITT population)

		**All N = 35**	**Masitinib N = 27**	**Placebo N = 8**
**Age** (years)	Mean ± SD	48 ± 8	49 ± 9	47 ± 7
	Min - Max	29–61	29–61	33–56
**Weight** (kg)	Mean ± SD	69 ± 19	67 ± 19	74 ± 20
	Min - Max	43–140	43–140	54–108
**Male**	N (%)	17 (49)	13 (48)	4 (50)
**Duration of disease** (years)	Mean ± SD	9.4 ± 7.4	9.5 ± 7.3	8.8 ± 8.4
	Min - Max	0.2-28.6	0.2-28.6	1.5-25.6
**MSFC score**	Mean ± SD	0.0 ± 0.7	-0.1 ± 0.7	0.3 ± 0.8
9-HPT (seconds)	Mean ± SD	30 ± 9	30 ± 9	31 ± 12
PASAT-3” (correct answers)	Mean ± SD	31 ± 15	30 ± 15	36 ± 15
T25FW^*^	Mean ± SD	N/A	N/A	N/A
**EDSS score**	Mean ± SD	4.9 ± 1.2	4.9 ± 1.2	5.0 ± 1.1

^*^ The baseline average of the raw (seconds) timed 25-foot walk test was not applicable (N/A) because of the protocol deviation (see text for details). *MSFC* = multiple sclerosis functional composite score. *N* = number of patients, intent-to-treat population. *T25FW* = timed 25-foot walk test. *9-HPT* = nine ­hole peg test. *PASAT-3”* = Paced Auditory Serial Addition Test 3 seconds. *EDSS* = expanded disability status scale. *ITT* = intent-to-treat.

### Safety analysis

Assessment of safety was performed on all patients who had received at least one treatment dose (i.e. the ITT population, N = 35), for the duration of treatment including any extension period until unblinding. The proportion of patients reporting at least one AE regardless of causality was similar between groups; specifically 22/27 masitinib treated patients (82%), of which only 19/27 patients (70%) were suspected to have experienced a treatment-related AE, and 6/8 placebo patients (75%). A summary of safety is presented in Table 2. The majority of AEs were of mild-to-moderate intensity and transitory, with the most frequent AEs observed in patients receiving masitinib being: asthenia (11/27 patients, 41%), rash (7/27, 26%), nausea (6/27, 22%), edema (5/27, 19%), and diarrhea (3/27, 11%). The frequency of severe AEs suspected to be treatment-related (or not assessable), was 7/27 patients (26%), with only rash recorded at a frequency >5% (2/27 patients, 7%). Consistent with the known safety profile of masitinib, hematological assessments showed a higher occurrence of events (≥10% difference between treatment groups) for masitinib treated patients as compared with placebo patients for: leucopenia, 6/27 patients [22%] versus none, respectively; and lymphopenia, 4/27 patients (15%) versus none. In the masitinib group there was one severe case (4%) of neutropenia reported, however, the majority of other hematological events were of mild intensity (70%). No severe decreases in white blood cell count, hemoglobin count, platelet count, or lymphocyte count were reported.

**Table 2:** Summary of safety data at time of unblinding (ITT population)

**Number (%) of patients with**	**Masitinib (N = 27)**	**Placebo (N = 8)**
**At least one AE** (all)	22 (81.5%)	6 (75.0%)
**At least one AE** (masitinib related)	19 (70.4%)	N/A
**Severe AE** (all)	8 (29.6%)	1 (12.5%)
**Severe AE** (masitinib related)	7 (26.0%)	N/A
**Serious AE** (all)	9 (33.3%)	2 (25.0%)
**Serious AE** (masitinib related)	4 (14.8%)	N/A
**Death** (all)	0 (0.0%)	0 (0.0%)
**AE leading to permanent discontinuation** (all)	7 (26.0%)	0 (0.0%)

*All* = regardless of causality. Masitinib related = suspected or not assessable. *N* = number of patients, intent-to-treat population. *N/A* = not applicable. *ITT* = intent-to-treat.

The median patient exposure to masitinib was 444 days (range 12–631 days). At the cut-off date of study unblinding, a total of 7/27 patients (26%) from the masitinib group had exited the study due to AEs compared with none from the placebo group. Of these, 3/27 patients (11%) had reported severe AEs, including decreased neutrophil count (onset after 27 days of treatment with duration of 15 days), urticaria (onset after 17 days with duration of 14 days), and hand-foot syndrome (Palmar-Plantar Erythrodysesthesia) (onset after 31 days with duration of 18 days). All cases resolved rapidly upon treatment discontinuation. No deaths were reported.

### Efficacy analysis

Unless stated otherwise, efficacy data from the mITT population according to the LOCF dataset are presented hereafter. A summary of response data at month-12 is presented in Table 3. At month 12 the average of the relative change in MSFC score with respect to baseline was +103% ± 189 (n = 24) versus −60% ± 190 (n = 6) in the masitinib and placebo groups, respectively. This positive, albeit non-statistically significant response was observable as early as month-3 and sustained through to month-18 (see Additional file 2: Details of masitinib response data). A total of 7/22 (32%) assessable masitinib patients (i.e. according to the observed cases dataset) reported clinical response following 12 months of treatment compared with none in the placebo group. The masitinib treated responders consisted of 2/8 (25%) PPMS patients and 5/14 (36%) rfSPMS patients.

**Table 3:** Summary of response at month 12 with subgroup analysis according to type of disease and MSFC subcategories (mITT population, LOCF method)

		**Placebo**	**Masitinib**		
		**All (n = 6)**	**All (n = 24)**	**PPMS (n = 9)**	**SPMS (n = 15)**
Relative change in MSFC score_*_	Mean ± SD	-60% ± 190	103% ± 189	134% ± 268	84% ± 130
Relative change in T25FW	Mean ± SD	26% ± 55	5% ± 26	13% ± 17	-1% ± 29
Relative change in 9-HPT	Mean ± SD	0% ± 13	-7% ± 9	-5% ± 7	-8% ± 10
Relative change in PASAT-3”	Mean ± SD	24% ± 30	41% ± 111	19% ± 66	55% ± 131
**Absolute change in EDSS score**	Mean ± SD	0.3 ± 1.0	0.0 ± 0.5	0.1 ± 0.4	0.0 ± 0.5

* Change from to baseline. In the primary endpoint of MSFC an increase represents an improvement in MS-related impairment. *mITT* = modified intent to treat. *LOCF* = last observation carried forward. *MSFC* = multiple sclerosis functional composite score. *T25FW* = timed 25-foot walk test. *9-HPT* = nine ­hole peg test. *PASAT-3”* = Paced Auditory Serial Addition Test 3 seconds. *EDSS* = expanded disability status scale.

The increase from baseline in MSFC for the overall population was mainly driven by T25FW and 9-HPT scores (see Additional file 2: Details of masitinib response data). The mean relative change in T25FW tended to increase over the duration of treatment indicating deterioration in performance; however, this was milder in the masitinib group as compared with the placebo group (i.e. 5% ± 26 versus 26% ± 55 at month 12, respectively). The relative change in 9-HPT tended to decrease in the masitinib group over the duration of treatment, indicating improved function from baseline, whereas no improvement was observed in the placebo group (i.e. -7% ± 9 versus 0% ± 13 at month 12, respectively; corresponding to absolute changes of approximately -2.2 versus 0.3 seconds, respectively). The relative change in PASAT-3” tended to increase throughout the study in both treatment groups (e.g. 41% ± 111 versus 24% ± 30 at month 12 in the masitinib and placebo groups, respectively; corresponding to absolute mean changes of approximately 5 versus 7 correct answers, respectively).

Overall, EDSS scores remained stable throughout the study in both treatment groups, with a mean change lower than 0.5 in EDSS (see Additional file 2: Details of masitinib response data). When analyzed by clinical course, EDSS score at month 12 was stable in the PPMS population in both treatment groups, whereas in the rfSPMS population EDSS score remained stable in the masitinib group but had increased in the placebo group by +1 point; an increase indicating deteriorating patient function.

## Discussion

Similar overall safety profiles were observed between the masitinib and placebo groups, although there was a higher incidence of severe and serious AEs associated with masitinib treatment. The most frequent masitinib-associated AEs were consistent with the known safety profile of tyrosine kinase inhibitors, notably rash, nausea, edema, and diarrhea, which are generally considered manageable with symptomatic treatments when of non severe intensity. The majority of AEs leading to permanent discontinuation in the present study were of non severe intensity, suggesting therefore a fairly cautious investigator approach to AEs or difficulties experienced in their management. As rash was the leading cause of discontinuation in this and other non-oncology masitinib trials (data not shown), future studies might consider consulting a dermatologist on matters of rash management and possible treatment interruption or dose adjustment prior to any decision on discontinuation.

Although efficacy data did not produce statistically significance differences between treatment groups, it does suggest a positive effect of masitinib on MS-related impairment and potential retardation of disease progression for both PPMS and rfSPMS patients. For example, in patients treated with masitinib we observed an improvement in MSFC scores relative to baseline, compared with a worsening MSFC score in patients receiving placebo. These changes were mainly driven by the T25FW and 9-HPT subscores, with the clinical implications being that masitinib might slow down the degeneration of lower limb function (as evidenced by a milder deterioration of T25FW) and improve upper limb function (as evidenced by improvement in 9-HPT). However, no adjustments were made for learning effects associated with some of the MSFC component measures, which may therefore have influenced these findings [19]. Also, for progressive diseases such as PPMS, the use of LOCF analysis is inclined to underestimate functional deterioration. Conversely however, considering the number of positive MSFC clinical responses achieved by masitinib patients (32%) compared with placebo patients (0%), it is unlikely that such effects had a major impact on the overall results.

Initially, 35 patients were planned for a treatment period of 36 months; however, this was amended to at least 20 patients who had completed at least 12 months of treatment. This protocol amendment, which effectively unblinded the study early, was implemented in part because even under blinded conditions it was probable that some masitinib-treated MS patients were among those showing positive response. In view of the pressing medical need for an effective treatment in progressive forms of MS, if this were the case then the primary objective to demonstrate acceptable safety and possible therapeutic response, i.e. establish proof-of-concept, would have been sufficiently accomplished, thereby enabling progression to the next development stage (i.e. phase 2b/3). One negative consequence of this reduced study population however, given the final dataset, was that it precluded any demonstration of statistical significance between the masitinib and placebo treatment. A second factor in the decision to amend the study population size was due to design factors and minor protocol deviations that would have complicated any definitive interpretation of efficacy, even if statistical significance had been demonstrated. This included a study amendment to close the 3.0 mg/kg/day treatment arm because of lack of response, effectively pooling all patients into the 6.0 mg/kg/day treatment arm. Also, it became apparent that there was a minor protocol deviation in the timed 25-foot walk (T25FW) test measuring leg function and ambulation, which forms part of the MSFC composite score. This test was misunderstood by two test centers representing 7 and 10 patients of the mITT population, respectively. One conducted the test on 25 steps and the other on 25 meters instead of 25 feet. The resultant disparity between centers was statistically compensated for by individually calculating each subpopulation’s T25FW z-score (i.e. with respect to units of steps, meters or feet) with reference to its overall patient average, and then taking the average of these z-scores for the overall T25FW z-score. This protocol deviation is expected to have had little or no effect on the interpretation of the MSFC score because the z-score (or standard score, a dimensionless quantity indicating how many standard deviations an observation is above or below the mean) allows direct comparison of observations from different units of measure.

The possible mechanisms of action by which masitinib may be capable of inducing the observed positive therapeutic response in patients with progressive MS are multifaceted. Although a topic of debate, there is growing evidence that the different courses of MS, i.e. relapsing as opposed to relapse-free, are due to distinct pathophysiologic processes. That is, RRMS and SPMS are probably different stages of the same disease while PPMS may imply different processes. Relapses are considered the clinical expression of acute inflammatory focal lesions whereas progression is considered to reflect the occurrence of demyelination, axonal loss and gliosis [20]. This distinction in MS types appears to be reflected by the unsuccessful treatment of PPMS with powerful disease modifying drugs. In turn, this may relate to the dominant cause of progression of disability in PPMS being more strongly related to nerve cell death, in addition to inflammation-induced neuronal damage (swelling) commonly attributed to relapsing forms of MS. As mentioned previously, there is good evidence in support of mast cells being actively involved in the pathogenesis of MS [12,13]. For example, sites of inflammatory demyelination contain cellular infiltrates with mast cell accumulation in the brain and spinal cord, [21] and the percentage of degranulated mast cells in the central nervous system correlates with the clinical onset of disease symptoms in acute EAE [22]. The contribution of mast cells to the pathological cascade of MS is in part because they release large amounts of proinflammatory mediators and therefore play a prominent role in sustaining the inflammatory network of the central nervous system [23]. The involvement of inflammation in the development of brain injury in MS is well-established, neurodegeneration being provoked in part by soluble inflammatory mediators, with a significant correlation existing between inflammation and acute axonal injury [12]. Moreover, perivascular mast cells secrete pro-inflammatory and vasoactive molecules that can regulate the BBB’s permeability, a defective BBB being a common finding that precedes clinical or pathological signs of MS [14,24,25]. Additionally, it has been shown in vitro that mast cell activation can lead to neuronal damage by inducing astroglia to produce neurotoxic quantities of nitric oxide (NO) [26]; NO being a molecule implicated in the pathogenesis of MS, especially for those patients in progression [27,28]. It has also been reported that mast cells can be a source of NO derivatives, which they synthesize spontaneously or following activation, depending on their subtype [29]. This evidence supports the notion that mast cells, which can be found in close vicinity to neurons, could influence the survival and functions of NO-sensitive cells and through this mechanism participate in the pathophysiology of chronic neurodegenerative diseases of the nervous system. Additionally, it is plausible that masitinib’s inhibitory action also effects the activation of dendritic cells, which are integral to the differentiation of T helper cells and regulate T cell responses, through inhibition of c-Kit and Lyn [30,31]. This hypothesis may be of significance as recent genetic findings particularly implicate T helper cell differentiation in the pathogenesis of MS [32].

## Conclusions

Thus, masitinib’s anti-mast cell properties, and possible effect on dendritic cells, may be particularly well adapted to the treatment of PPMS. A reduction of mast cell activity via the inhibitory action of masitinib on c-Kit, Lyn and Fyn tyrosine kinase activity, impacting both inflammatory mediated and NO-mediated damage mechanisms, while inhibition of dendritic cell activity may disrupt the signaling pathways relevant to T helper cells. The findings of the current study, within limitations inherent to such an exploratory trial, suggest that oral masitinib was relatively well tolerated and can be of therapeutic potential in the treatment of MS, with positive responses observed in some relevant measures of this condition. Moreover, this positive action was observed in patients with PPMS and rfSPMS, subpopulations for whom there are practically no currently available treatments. Taken together with positive results from the complementary EAE mouse model, this trial provides evidence that supports a larger placebo-controlled investigation.

## Competing interests

Masitinib is under clinical development by the study sponsor, AB Science (Paris, France). AM is an employee and shareholder of the study sponsor. OH and PD are consultants and shareholders of the study sponsor. No other conflicts of interest have been declared.

## Authors’ contributions

PV was the principal clinical investigator who contributed to the study’s conception and design, patient recruitment and treatment, data analysis and interpretation, and manuscript preparation. RB, NS, HZ, PC and CV were clinical investigators who contributed to the recruitment and treatment of the patients. AM, PD and OH contributed to the study’s conception and design, data analysis and interpretation, and manuscript preparation. All authors critically reviewed the manuscript and gave final approval of the version to be published.

## Pre-publication history

The pre-publication history for this paper can be accessed here:

http://www.biomedcentral.com/1471-2377/12/36/prepub

## Supplementary Material

Additional file 1 **Preclinical data of masitinib in EAE.** Preclinical data of masitinib in a myelin oligodendrocyte glycoprotein (MOG)-induced experimental allergic encephalomyelitis (EAE) murine model. Click here for file

Additional file 2 **Details of masitinib response data.** Details of masitinib response data - mean percentage change from baseline at months 3, 6, 9, 12, and 18 (mITT population). Click here for file
