# Supplementary material for: Masitinib treatment in patients with progressive multiple sclerosis: a randomized pilot study
Source: BMC Neurol. 2012 Jun 12;12:36. doi: 10.1186/1471-2377-12-36 (PMC3467179; doi:10.1186/1471-2377-12-36)
Supplement: Additional file 1 — Preclinical data of masitinib in EAE. Preclinical data of masitinib in a myelin oligodendrocyte glycoprotein (MOG)-induced experimental allergic encephalomyelitis (EAE) murine model. [file 1471-2377-12-36-S1.pdf]

Additional Files

Additional file 1: Preclinical data of masitinib in EAE

Preclinical data of masitinib in a myelin oligodendrocyte glycoprotein (MOG)-induced experimental allergic encephalomyelitis (EAE) murine model.

Supplemental information for the article:

Masitinib treatment in patients with progressive multiple sclerosis: Results of an exploratory phase 2a clinical study. Patrick Vermersch, Rabah Benrabah, Nicolas Schmidt, Pierre Clavelou, Hélène Zéphir, Cyrille Vongsouthi, Patrice Dubreuil, Alain Moussy and Olivier Hermine.

Additional file 1: Preclinical data of masitinib in EAE

Introduction

It has been reported that in the context of the experimental allergic encephalomyelitis (EAE) model, an animal model of human demyelinating diseases including multiple sclerosis (MS), mast cells are necessary for the full manifestation of myelin oligodendrocyte glycoprotein (MOG)-induced EAE disease [11-14]. Increased permeability of the Blood-Brain Barrier is an early event in the development of clinical or pathologic findings in EAE and MS with perivascular brain mast cells secreting vasoactive and pro-inflammatory molecules then contribute to the pathological cascade [24,25]. Sites of inflammatory demyelination contain cellular infiltrates with mast cell accumulation in the brain and spinal cord [21], and the percentage of degranulated mast cells in the central nervous system correlates with the clinical onset of disease symptoms in acute EAE [22]. Furthermore, mast cell-deficient mice exhibit significantly reduced disease severity compared to wild-type littermates in a murine model of MS and drugs that block mast cell function can improve clinical symptoms in this model.

Methods

The effect of masitinib in the inhibition of mast cell function in MS was explored using the EAE murine model. The MOG-induced EAE disease in C57BL/6 mice is considered to be a model for all progressive forms of MS. C57BL/6 mice were immunized with 300 ng MOG35-55 peptide (an immunological target in the human disease) on day 0 (in complete Freund’s adjuvant) and day 7 (in incomplete Freund’s adjuvant) and 250 ng pertussis was administered intravenously on days 0 and 2. Mice were scored daily by visual assessment of symptoms on a scale of 0-5 where: 1 denotes a flaccid tail; 2 denotes hind limb weakness; 3 denotes hind limb paralysis; 4 denotes an inability to right from supine; and 5 indicates death. Statistical analyses of significance between mean clinical scores were performed using Bonferroni's Multiple Comparison Test-One way ANOVA or paired t-tests. Statistical analyses were performed for the daily intervals between days 26-35. Five mice per

experimental group were administrated the following treatment every day from the first day of immunization (day 0):

- Vehicle (PBS)
- Masitinib 25 mg/kg, 2 times per day, intraperitoneal injection
- Masitinib 12.5 mg/kg, 2 times per day, intraperitoneal injection

Results

Between days 26-35, treatment with masitinib at both doses produced significant differences in mean clinical score (Figure S1). A dose response between 25 mg/kg and 12.5 mg/kg was also observed.

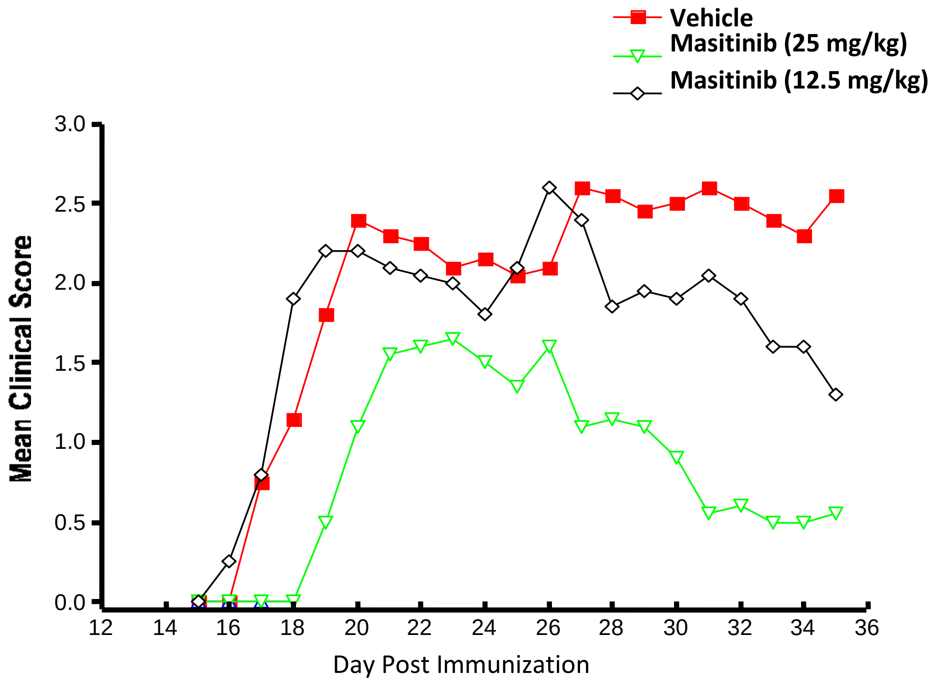

Figure S1: Effect of masitinib on the clinical score in the mouse EAE model of MS

Vehicle versus masitinib 25 mg/kg	p< 0.001
Vehicle versus masitinib 12.5 mg/kg	p< 0.001
Masitinib 25 mg/kg versus masitinib 12.5 mg/kg	p< 0.001

Conclusions

Treatment of mice with masitinib led to a significant reduction in disease, as assessed by the mean clinical score, when compared with mice treated with vehicle alone. There appears to be a masitinib dose-dependent effect, and also a delayed response to masitinib at both doses.
