# Supplementary material for: Masitinib treatment in patients with progressive multiple sclerosis: a randomized pilot study
Source: BMC Neurol. 2012 Jun 12;12:36. doi: 10.1186/1471-2377-12-36 (PMC3467179; doi:10.1186/1471-2377-12-36)
Supplement: Additional file 2 — Details of masitinib response data. Details of masitinib response data - mean percentage change from baseline at months 3, 6, 9, 12, and 18 (mITT population). [file 1471-2377-12-36-S2.pdf]

Additional Files

Additional file 2: Details of masitinib response data

Details of masitinib response data - mean change from baseline at months 3, 6, 9, 12, and 18 (mITT population, LOCF method).

Supplemental information for the article:

Masitinib treatment in patients with progressive multiple sclerosis: Results of an exploratory phase 2a clinical study. Patrick Vermersch, Rabah Benrabah, Nicolas Schmidt, Pierre Clavelou, Hélène Zéphir, Cyrille Vongsouthi, Patrice Dubreuil, Alain Moussy and Olivier Hermine.

Additional file 2: Details of masitinib response data - mean change from baseline at months 3, 6, 9, 12, and 18 (mITT population, LOCF method).

			Month 3	Month 6	Month 9	Month 12	Month 18
**MSFC score** Relative change (%) (Mean ± SD)	All (n = 30)	Masitinib (n = 24)	78 ± 284	156 ± 274	70 ± 267	103 ± 189	96 ± 191
		Placebo (n = 6)	-64 ± 188	-58 ± 200	-49 ± 193	-60 ± 190	-61 ± 190
	PPMS (n = 12)	Masitinib (n = 9)	108 ± 435	202 ± 353	56 ± 410	134 ± 268	134 ± 268
		Placebo (n = 3)	-14 ± 40	-20 ± 92	8 ± 21	-11 ± 47	-11 ± 47
	SPMS (n = 18)	Masitinib (n = 15)	60 ± 152	129 ± 223	78 ± 146	84 ± 130	73 ± 131
		Placebo (n = 3)	-113 ± 281	-96 ± 296	-106 ± 287	-109 ± 284	-110 ± 284
**T25FW** Relative change (%) (Mean ± SD)	All	Masitinib	-2 ± 12	-1 ± 24	9 ± 31	5 ± 26	6 ± 26
		Placebo	19 ± 58	17 ± 60	29 ± 53	26 ± 55	37 ± 52
	PPMS	Masitinib	1 ± 9	12 ± 16	21 ± 34	13 ± 17	13 ± 17
		Placebo	1 ± 7	-1 ± 16	8 ± 3	13 ± 3	13 ± 3
	SPMS	Masitinib	-4 ± 13	-9 ± 26	2 ± 28	-1 ± 29	2 ± 30
		Placebo	37 ± 87	34 ± 89	49 ± 76	39 ± 85	61 ± 70
**9-HPT** Relative change (%) (Mean ± SD)	All	Masitinib	-4 ± 9	-6 ± 11	-6 ± 12	-7 ± 9	-5 ± 10
		Placebo	2 ± 13	0 ± 19	-1 ± 16	0 ± 13	3 ± 13
	PPMS	Masitinib	-6 ± 10	-7 ± 8	-4 ± 12	-5 ± 7	-6 ± 7
		Placebo	6 ± 11	5 ± 19	2 ± 11	2 ± 9	2 ± 9
	SPMS	Masitinib	-4 ± 8	-6 ± 13	-8 ± 12	-8 ± 10	-4 ± 12
		Placebo	-1 ± 17	-5 ± 21	-5 ± 22	-2 ± 18	3 ± 19
**PASAT-3”** Relative change (%) (Mean ± SD)	All	Masitinib	31 ± 107	43 ± 104	38 ± 106	41 ± 111	44 ± 111
		Placebo	18 ± 26	28 ± 43	25 ± 29	24 ± 30	36 ± 61
	PPMS	Masitinib	20 ± 69	14 ± 58	15 ± 65	19 ± 66	16 ± 65
		Placebo	1 ± 10	5 ± 15	9 ± 3	7 ± 4	7 ± 4
	SPMS	Masitinib	37 ± 127	60 ± 123	51 ± 125	55 ± 131	60 ± 131
		Placebo	36 ± 26	51 ± 53	41 ± 36	41 ± 36	65 ± 81
**EDSS** Absolute change (Mean ± SD)	All	Masitinib	-0.1 ± 0.3	-0.1 ± 0.4	0 ± 0.4	0 ± 0.5	0 ± 0.5
		Placebo	0.1 ± 0.4	0 ± 0.8	0.1 ± 0.8	0.3 ± 1.0	0.3 ± 1.0
	PPMS	Masitinib	-0.1 ± 0.2	-0.2 ± 0.5	-0.1 ± 0.4	0.1 ± 0.4	0.1 ± 0.4
		Placebo	0 ± 0.4	-0.3 ± 0.8	-0.2 ± 0.8	-0.2 ± 0.8	-0.2 ± 0.8
	SPMS	Masitinib	0 ± 0.3	0 ± 0.3	0 ± 0.3	0 ± 0.5	0 ± 0.5
		Placebo	0.3 ± 0.6	0.5 ± 0.5	0.7 ± 0.6	1.0 ± 1.0	1.0 ± 1.0

In the primary endpoint of MSFC an increased score relative to baseline represents an improvement in MS-related impairment. Increased scores represent deterioration in the 9-HPT, T25FW and EDSS. Decreased scores represent deterioration in the PASAT-3 and EDSS. mITT = modified intent to treat population. LOCF = last observation carried forward. MSFC = multiple sclerosis functional composite score. T25FW = timed 25-foot walk test. 9-HPT = nine hole peg test. PASAT-3” = Paced Auditory Serial Addition. Test 3 seconds. EDSS = expanded disability status scale.
